# The Flux-Based PIN Allocation Mechanism Can Generate Either Canalyzed or Diffuse Distribution Patterns Depending on Geometry and Boundary Conditions

**DOI:** 10.1371/journal.pone.0054802

**Published:** 2013-01-28

**Authors:** Michael Luke Walker, Etienne Farcot, Jan Traas, Christophe Godin

**Affiliations:** 1 INRIA, Virtual Plants Project Team, UMR AGAP, Montpellier, France; 2 Laboratoire de Reproduction et Développement des Plantes, ENS-Lyon, CNRS, INRA, Université Claude Bernard, Lyon, France; Centrum Wiskunde & Informatica (CWI) & Netherlands Institute for Systems Biology, The Netherlands

## Abstract

Growth and morphogenesis in plants require controlled transport of the plant hormone auxin. An important participant is the auxin effluxing protein PIN, whose polarized subcellular localization allows it to effectively transport auxin large distances through tissues. The flux-based model, in which auxin flux through a wall stimulates PIN allocation to that wall, is a dominant contender among models determining where and in what quantity PIN is allocated to cell walls. In this paper we characterise the behaviour of flux-based PIN allocation models in various tissues of the shoot apical meristem. Arguing from both mathematical analysis and computer simulations, we describe the natural behaviours of this class of models under various circumstances. In particular, we demonstrate the important dichotomy between sink- and source- driven systems, and show that both diffuse and canalized PIN distributions can be generated simultaneously in the same tissue, without model hybridization or variation of PIN-related parameters. This work is performed in the context of the shoot apical and floral meristems and is applicable to the construction of a unified PIN allocation model.

## Introduction

The plant hormone auxin plays a major role in plant growth and morphogenesis. The dynamic allocation of the auxin effluxing protein PIN is an integral part of this process. It allows developing meristems to determine the position of the next primordium, where organs grow, and to define the path of vascular strands within the tissue.

**Figure 1:**
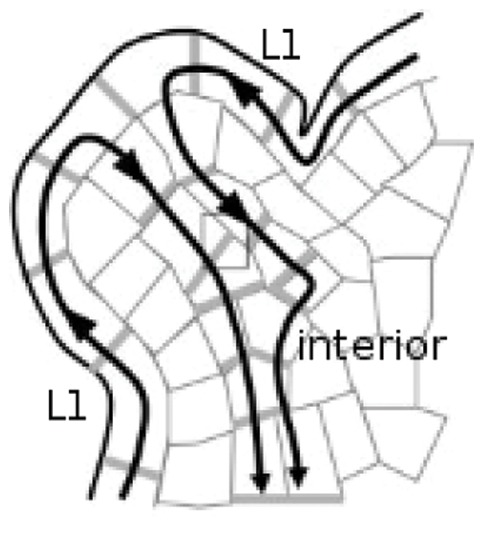
Cutaway illustration of shoot apical meristem. PIN concentrations are indicated by grey, while the black arrows show the direction of auxin flux. The auxin uxes towards the primordium within the L1 surface layer, where it is ushed into the interior. Hence the primordium is an effective sink in the L1, removing auxin from it, but forms an effective source for the interior of the meristem since the auxin enters the interior through the primordium. Adapted with minor modification from figure 1B in [31].

Above-ground growth and development in a plant takes place in the shoot apical meristems (SAMs). These are located at the tip of the shoots and maintain at their centre a central zone of undifferentiated stem cells which continuously divide and grow in a way that gradually displaces them outwards, until they leave the central zone and enter the peripheral zone. Here they differentiate into specific cell types. A select few become primordial cells and begin the development of new organs. This process requires, and may even be initiated by, the polarized localization of PIN in surrounding cells in the surface layer (L1) towards the new primordium [1], [2], which consequently receives a large influx of auxin. As shown in figure 1, the primordium then transports this auxin into the interior of the SAM via PIN-mediated transport, where it stimulates the formation of a canalized PIN distribution (vascular strand) and lays the path for a future vein. This dualistic expression pattern requires a model that forms canals in the interior of the SAM but not on its surface. Finally, the evacuation of auxin from the L1 creates a region whose cells have a relatively low concentration of auxin around the primordium, called a *depletion zone*. This lack of auxin is believed to inhibit the formation of other primordia in this zone, which leads to the generation of phyllotactic patterns.

Understanding how the cells decide where and how much PIN to express is therefore essential to understanding plant organ development. Several different models have been proposed, the prominent ones being the ``flux-based` ` model [3], [4], [5] and the “concentration-based” model [6], [7].

The concentration-based model allocates PIN in the cell wall according to the auxin level of the adjoining cell. This creates a positive feedback loop by enhancing peaks that are already present, which makes it a natural candidate for generating auxin maxima. Indeed, van Mourik *et al.* [8] applied this model to a digitized floral meristem (FM) and successfully produced auxin peaks in positions that approximately correspond to those of incipient petals and stamens, while [9] found experimental evidence for a mechanism by which the auxin concentration of a cell can feed back on the PIN localization in its neighbours, by altering that cell’s mechanical properties and the stresses induced on the neighbours. Unfortunately the concentration-based model is a poor candidate for vascular strand formation, except through a “travelling pulse” mechanism [10]. This mechanism considers the experimental fact that PIN production is sensitive to auxin concentration which creates the moving auxin pulse at the cost of rendering stationary auxin peaks unstable. More seriously, the approach of [10] is inconsistent with experimental observations that the auxin peak remains and increases at the primordia [11].

The flux-based model allocates PIN to a cell face according to the total amount of flux that is passing through it, creating a positive feedback loop by enhancing transport through walls that already have a significant flux through them. Not only is this an effective mechanism for growing canalized PIN formations [12], but it can also lead to the formation of auxin peaks in and around cells that degrade or otherwise remove auxin from the tissue (sinks) [5].

Stoma *et al.* [5] changed the PIN distribution from one that forms canals to one that converges on the sink by switching the feedback function from quadratic to linear. The contrasting approach of Bayer *et al.* [13] was a hybrid model, in which every cell runs both the flux-based and concentration-based models in a combination determined by its auxin concentration. At low auxin concentrations, cells favour the concentration-based model to generate the auxin maximum in the primordium. At higher concentrations they favour the flux-based model, thus generating the vascular strand connecting the primordium to the vein draining the SAM. Their paper predicted new PIN polarization dynamics within the SAM. Neither of these papers discusses the possibilty of non-canalized PIN distributions arising from a quadratic flux-based model.

Although they frequently coincide, convergent PIN patterns are distinct from auxin peaks. We discuss the former more frequently than the latter in this paper due to our particular interest in PIN dynamics, although the occurence of auxin peaks is of particular relevance to phyllotaxis. In concentration-based models the auxin peak generates the convergent PIN pattern [6], [7], while in flux-based models convergent PIN patterns can generate auxin peaks. This is important because convergent PIN patterns typically form around sinks, leading to the counter-intuitive formation of auxin peaks at sinks, as first observed by Stoma *et al.* [5] and also shown in the results of this paper.

In this paper we have demonstrated that the two PIN distributions, canalized (or canal forming) and convergent to use Stoma’s terminology [5] can be generated by one single model and one set of parameters. We have generalised the discussion of convergent PIN allocations to include distributions diverging outward from a source and refer to these in general as being *diffuse*. The occurence of either canalized or diffuse PIN distributions in different parts of the SAM is found to arise from inherent differences in the source/sink distribution between the L1 and the interior. Indeed, the SAM and the FM can both be dissociated into source-driven and sink-driven systems as illustrated in figure 1. The L1, being composed of sinks, the primordia, surrounded by a continuous source, consists of “sink-driven” systems, while the interior, for which the primordia act like sources effluxing into a tissue that otherwise produces little or no auxin, consists of “source-driven” systems. We found that canalization in these two systems exhibits markedly different sensitivities to various factors, including cell shape and regularity, and the exponent of the feedback function, allowing them to simultaneously show different PIN distributions with the same model and parameters. We have demonstrated this on a variety of tissue geometries including some digitized ones. We performed simulations in both two and three dimensions and have discussed when and how well a three-dimensional simulation can be approximated by a two-dimensional simulation. We found that the result in three dimensions can sometimes be qualitatively different from that in two dimensions.

Table 1 illustrates our main finding, which was supported by both analytic arguments and computer simulations. It was that canalized configurations are very robust for source-driven systems but are not very robust for sink-driven systems.

**Table 1:** Factors affecting canalization.

insensitive to	*σ*	parameters	2D vs 3D	cell geometry
polar-in	N	N	N	N
polar-out	Y	suff. auxin flux	Y	Y
sink-driven canalization	N	N	2D	N
source-driven canalization	Y	Y	Y	Y
strand straightness	Y	Y	Y	flux density

**Summary of main results.** The feedback exponent 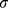 is taken to be greater than one since the linear case never yields canalization. Values other than Y(es) or N(o) indicate necessary conditions.

## Models

### Model Description

With the exception of sinks and sources, each cell has a background auxin generation rate 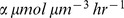 and degradation rate 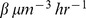, so that auxin concentration in a given cell varies as
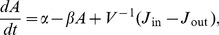
(1)where 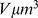 is the cell volume, equal to one 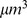 in our simulations unless stated otherwise, and 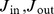 represent the auxin flux in 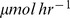 into and out of the cell, respectively.

Auxin transport between cells is effected by passive bidirectional permeation of cell walls (we neglect the apoplastic chamber), and active transport generated by auxin effluxing molecules called PIN. The flux from cell *i* to cell *j* is therefore
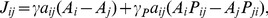
(2)where 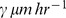 is the permeation constant of the cell wall, 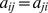 is the area 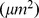 of the wall separating cells 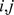. The auxin concentration of cell 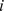 is denoted 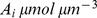, 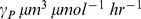 is the PIN transport coefficient and 
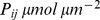
 is the density of PIN in the cell wall effluxing from cell 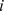 to cell 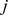. For cell 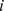 with neighbours labelled 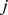 the relationship between 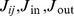 is given by
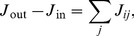
(3)where we employ the convention that positive 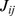 is directed out of the cell.

So far our equations are functionally identical to that used by other authors [4], [5], although some include saturation terms [4].

The PIN dynamics contain both a background PIN generation rate 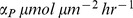, which in this paper equals zero unless stated otherwise, and PIN degradation rate 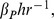 as well as a flux dependent contribution to the PIN generation rate which specifies the PIN model.

In addition to flux-based models, we also ran simulations of *flux-density* based models, models in which the flux 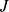 is replaced by the flux-density
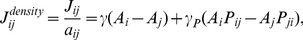
(4)which is the flux through wall 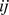 divided by the area of wall 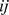. Furthermore, we considered these two mechanisms in both the noncompetitive model used by Stoma *et al.* [5] and Sachs [14], and in a simple competitive model, like that of Feugier *et al.* [4].

In the noncompetitive case PIN is generated in walls according to the flux passing through them. The PIN dynamics are described by
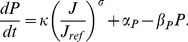
(5)

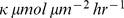
 is just a proportionality constant, while 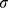 represents the exponent of the flux dependence.

A wall’s PIN generation in the competitive model is also flux dependent but the walls must compete from a limited pool. The PIN dynamics in this model are
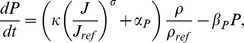


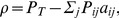
(6)where 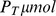 is the total amount of PIN available to the cell. (The corresponding equation in [4], equation (3b), is seen to be equivalent to this by substituting 
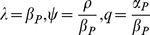
.) 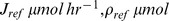 are used to simplify the units. We shall simplify notation henceforth by setting 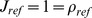.

Stoma *et al.* considered the non-competitive flux-based model described by equation (5) for 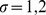 and found a switch from diffuse PIN distributions to canalized ones as one changes from the linear to the quadratic function. We have tried 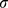 at various intermediate values. To avoid confusion with nonlinear equations with exponents of less than one we shall use the term *super-linear* to describe 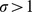 and reserve the term quadratic for 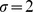.

The competitive model naturally contains a cut-off for the PIN concentration, and we impose one on the noncompetitive model, partly to contain numerical errors which Feugier *et al.* [4] also encountered, but also to respect the physical-biological fact that a plasma membrane of finite size can only contain a limited number of PIN molecules. Obviously when PIN concentrations are much lower than the cut-off then the cut-off has no effect, but it has large effects when PIN concentrations get high enough. We shall describe such effects as being due to saturation.

### Characterizing Canalized Systems

**Figure 2:**
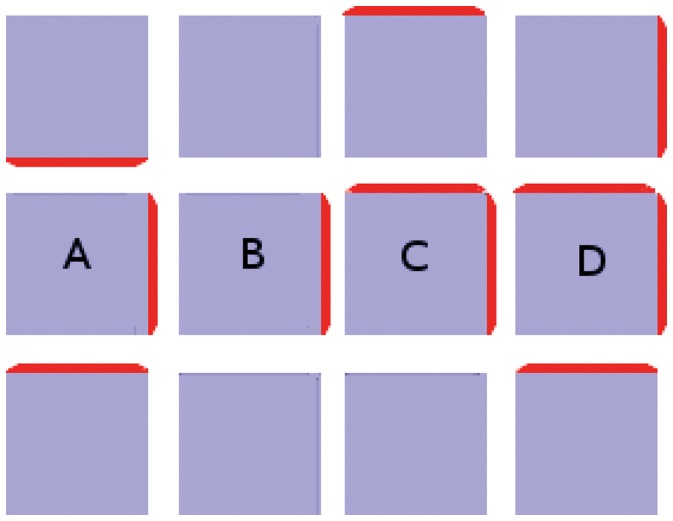
Cartoon illustration of terms polar-in, polar-out, multi-in and multi-out. (A) Cell A is multi-in and polar-out. (B) Cell B is polar-in and polar-out. (C) Cell C is polar-in and multi-out. (D) Cell D is multi-in and multi-out.

A strictly canalized PIN allocation obeys two conditions. The first is that any cell significantly expressing PIN must be unambiguously polarized in one direction. We call this the *polar-out requirement* (see figure 2 for an illustration of this and the following definitions), and the associated cell behaviour we call *polar-out behaviour*. The second is that no more than one of a cell’s neighbours may point a significant concentration of PIN toward it, which we call the *polar-in requirement*, and the associated behaviour *polar-in behaviour*. Failure to meet these requirements is referred to as *multi-out* and *multi-in behaviour*, respectively.

We use the term *source* to indicate a cell, or localized group of cells, which adds auxin to a tissue. In the same way, a *sink* is a cell, or localized group of cells, which removes auxin from a tissue. This functional definition can sometimes be counterintuitive as it is not uncommon for sinks to have a higher auxin concentration in and around them, even in flux-based models [5] which are capable of fluxing auxin against auxin gradients. Obviously the sink must be of limited efficiency, and we differentiate between *perfect* sinks, in which auxin is degraded immediately so that the cell’s auxin concentration is fixed at zero, and *realistic* sinks, in which auxin is degraded at a finite rate.

Sources can be similarly classified as being either *perfect*, having a fixed, non-zero auxin concentration, or *realistic*, producing auxin at a finite rate. It is likewise feasible for sources to have a relatively low concentration, according to whether there is sufficient PIN to remove auxin efficiently.

Although not acknowledged in the literature, the dynamics of flux-based simulations are usually driven by either sinks or sources. Models like those of Stoma *et al.* [5] are said to be *sink driven*, meaning that the dynamics are driven by a localized region, typically one or a few cells, which remove/destroy auxin much faster than the rest of the (approximately) uniform tissue. The top layer of cells in the shoot apical meristem, the L1, has dynamics of this type. There is auxin production throughout the L1 while the primordium forms an effective sink, removing it from the L1 and flushing it into the interior of the meristem [1], [2] (see figure 1). In the interior of the meristem a primordium forms an effective auxin source, since it is through the primordium that auxin enters the interior of the meristem. We say that the dynamics of the interior are those of a *source-driven* system, in which the dynamics are driven by a cell or localized group of cells which produce/efflux auxin much faster than the rest of the tissue. We found that whether the dynamics are source- or sink- driven has a large effect on whether a simulation obeys the polar-out and polar-in requirements.

### Materials and Methods

The digitized meristem and the digitized L1 were both generated using the MARS package [15]. Our simulation code relied heavily on the OpenAlea package (http://www-sop.inria.fr/teams/virtualplants/wiki/doku.php?id=software) developed by the INRIA project team Virtual Plants (http://www-sop.inria.fr/virtualplants/wiki/doku.php).

## Results

### Conditions for Polar-out Behaviour

We investigated the conditions for the model to demonstrate polar-out behaviour. A linear stability analysis of steady states was performed by Feugier [16] (appendix B). He analyzed the PIN dynamics of two identical walls in a cell in a noncompetitive flux-based model with quadratic dependence of the PIN generation on auxin flux as a function of the total amount of auxin either generated in or entering the cell. We designate this quantity
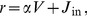
(7)where the reader is reminded that 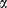 is the auxin biosynthesis rate per volume from equation (1). Feugier found that polar-out PIN allocation is the stable solution for 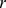 above the critical value 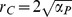, where we remind the reader that 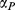 is the background PIN generation rate. We have found that the critical flux at which a cell with 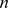 walls makes the transition to polar-out behaviour is 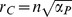. The mathematical derivation of this and Feugier’s result is presented in detail in text S1. Since our simulations took 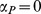, once the PIN-mediated flux became strong enough to dominate diffusion we always had polar-out behaviour in the steady state in the absence of saturation effects, described at the end of this section.

The second important result of this subsection is that the degree of super-linearity required for polar-out behaviour to be a stable outcome is vanishingly small. The mathematical derivation of this is presented in text S2 where we study the equations when 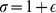 and find that 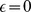 is a critical value. The analysis is only valid for low 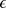 and the 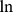 expansion requires 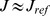, but higher order terms will only increase the super-linearity and are scarcely likely to restore the diffuse distribution.

These analyses neglected the effect of a PIN cut-off or a limited PIN supply. However we did use a cut-off in our non-competitive simulations, both to avoid numerical problems and to reflect the physical reality of finite wall areas and PIN sizes. We typically chose PIN cut-offs high enough to avoid interference with the flux-based dynamics, but if a cut-off was too low for one wall to efflux all the auxin coming into a cell then the PIN level saturated. The auxin level then rose, and if it did so sufficiently then another wall began to express PIN. If the auxin influx later dropped then it was the second, weaker PIN expression that degraded while the original, stronger PIN remained dominant, as expected from equation (5). Hence it is possible to have a limited build-up of auxin in a non-competitive model, but beyond that limit a second PIN builds up and flushes it away. More importantly, a PIN cut-off can generate exceptions to polar-out behaviour in a super-linear flux-based model. We shall see examples of this in the section on sink-driven systems.

The competitive model automatically imposes an effective cut-off, although its dynamics mimic those of the noncompetitive model when PIN concentrations are lower than the saturation regime. Super-linearity ceases to hold in the saturation regime so our arguments for polar-out behaviour break down. This is consistent with our simulations of the competitive model often converging on multi-out states.

In summary, polar-out behaviour is the default for super-linear flux-driven simulations, even if the degree of super-linearity is vanishly small. Both analysis and simulation found that it always holds in the absence of saturation effects.

### Conditions for Polar-in Behaviour

We discuss the factors affecting whether and how many of a cell’s neighbours allocate PIN to their mutual wall. Essentially, a neighbour will do so if it can deliver sufficient auxin flux. The auxin flux needed to generate PIN originates from both production within the cell and auxin flux received from neighbouring cells. If all auxin- and PIN- related parameters are equal throughout a tissue, then the difference in auxin flux into a given cell between two of its neighbours can be attributed to their receiving different auxin flux from other cells.

**Figure 3:**
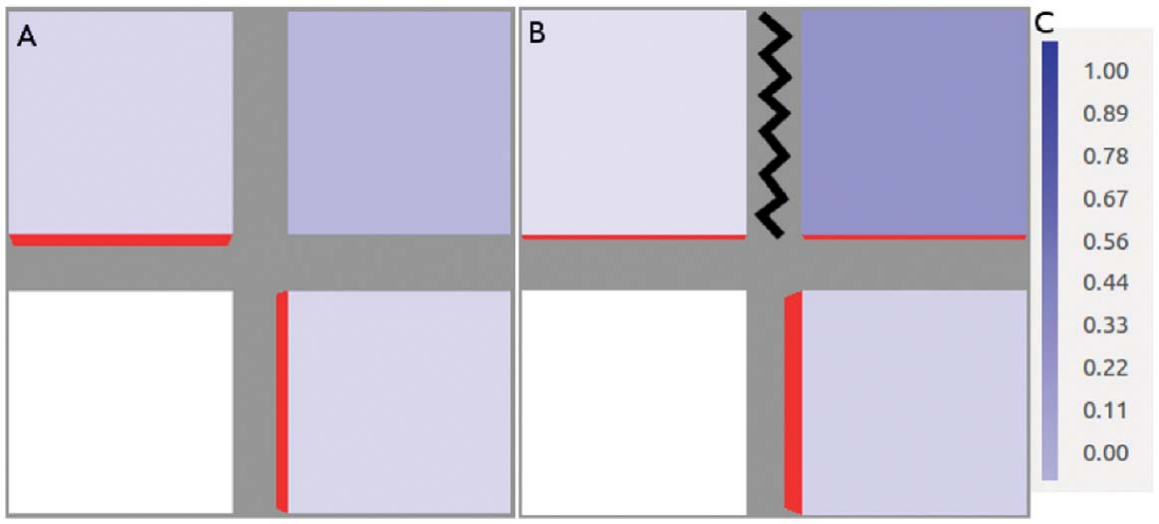
Demonstration of competition for flux between neighbouring cells. In both simulations the bottom left-hand cell is a perfect sink while all other cells generate auxin at a rate of 0*∶*4 *µmolhr^−^* ^1^. (A) Neighbours of the sink share the auxin effluxing from their mutual neighour in the top right-hand corner, so they each receive enough to generate a small concentration of PIN. They also receive identical PIN-mediated flux, even if the top left cell enjoys an initial advantage of 10*^−^* ^10^ *µmol* *µm^−^* ^2^ *;* indicating that identical PIN concentrations constitute the stable configuration. (B) Auxin transport between the top two cells is blocked, as indicated by the black zigzag. The top right-hand cell now fluxes auxin exclusively to its lower neighbour. This raises its total auxin efflux enough to express PIN strongly towards the sink, while the sink’s other neighbour has weaker PIN expression than its couterpart in (A). The feedback exponent is given by 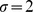. (C) This colour bar indicates the auxin concentration in *µmol* *µm^−^* ^3^ in this figure and the next one.

We illustrate this idea in figure 3. Both tissues have a perfect sink in the bottom left corner, while the cells generate and degrade auxin at fixed rates while running the flux-based model. (In this, as in all figures, auxin concentration is in 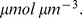) Where the two tissues vary is that the cell at top right feeds auxin to both its neighbours in the first picture but in the second it is barred from fluxing auxin to its left hand neighbour in any way. In the former case its auxin is shared with both of its neighbours, both of which receive only enough flux to generate small concentrations of PIN towards the sink. In the latter its auxin flows exclusively through one neighbour, which consequently has sufficient flux to generate a stronger PIN concentration (shown in red, where the thickness of the red bar indicates concentration), while the other neighbour’s PIN expression is weakened.

Competition is an important factor in the distribution of auxin flux. Cells receiving auxin from a common neighbour do not necessarily receive it in equal portions. The diffusive component of flux will go to the neighbour with the lowest auxin concentration, which will retain this majority of the diffusive flux if and only if it can maintain its low auxin concentration. Whether this happens follows from Feugier’s linear stability analysis. The receiving cell’s auxin throughput, which may originate in whole or in part from the shared neighbour, must be greater than the critical value 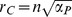 where 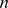 is the number of cell walls (see text S1). A cell receiving more net influx than the critical amount will outcompete a mutual neighbour that does not.

**Figure 4:**
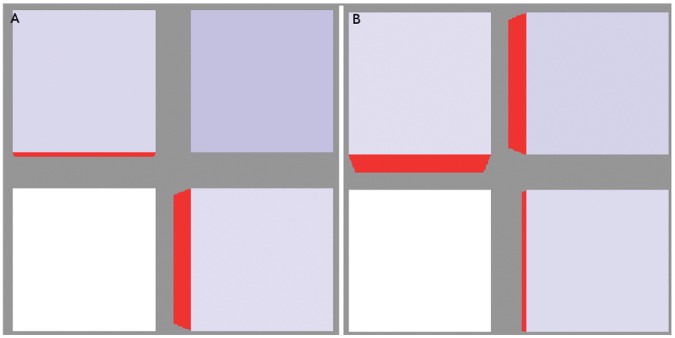
The two mechanisms of competition. (A) The auxin production rate in the bottom right cell has been increased to 2 *µmolhr^−^* ^1^ while the other auxin-producing cells both retain a production rate of 0.4 *µmolhr^−^* ^1^. This enhanced auxin throughput, via the increased PIN levels, maintains a low auxin concentration (0.4279 *µmol* *µm^−^* ^3^) that, paradoxically, gives it the majority of the diffusive transport. Since PIN in the top right cell (*A = *0.3727 *µmol* *µm^−^* ^3^) is too low to dominate transport this cell ends up receiving the majority of its ux. (B) Here it is the top right cell that has the enhanced auxin production (also 2 *µmolhr^−^* ^1^). In this case the initially tiny difference in PIN concentration between the two walls becomes very large, indicating that the flux through this cell is strong enough to make different PIN concentrations the stable configuration.

This is illustrated in figure 4A. Here the auxin production of the bottom right cell was increased to 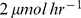 while the other auxin-producing cells both retained a production rate of 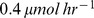. Its auxin throughput was sufficiently above the critical value to maintain a lower auxin concentration. It consequently received more diffusive flux than its competitor leading to a greater share of PIN-mediated flux from the top right cell.

The other factor in competition involves the PIN allocation within the common neighbour cell. While the PIN-mediated flux is less than the critical flux the PIN dynamics favour an even distribution. However, at greater than the critical flux the PIN distribution favours one particular wall over the others, as discussed in the section on polar-out behaviour. Thus, if any wall has a greater efflux than the others then the cell will polarise in that direction. This is demonstrated in figure 4B. Here the auxin production was elevated to 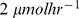 in the top right cell to give it a throughput greater than critical. The result was that the PIN distribution is strongly polarized towards one wall. Since the critical auxin flux marking the onset of polar-out behaviour is proportional to the number of cell walls, as shown in text S1 and described in the previous section, for a given flux throughput 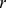 this mechanism is weaker when the common neighbour has more sides.

The relative importance of these two mechanisms will vary from one situation to another, but can be determined by adjusting either of the parameters 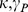. Increasing these parameters lowers the critical flux marking the onset of polar-out behaviour but increases the tendency of a cell to express PIN at all. Therefore, if increasing these parameters decreases polar-in behaviour then the first mechanism is more important, and vice versa. Performing simulations analogous to figure 3B in Stoma et al. [5], we observed a transition from diffuse to canalized by lowering 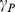, indicating that in these simulations it was competition for diffusive flux that dominated the dynamics. We shall say more about this in our discussion of sink-driven systems.

Our conclusion is that polar-in behaviour is sensitive to the availability and distribution of auxin flux. This in turn is sensitive to parameter values as well as cell topology and geometry. This last factor is important as the wall area obviously affects the transport rate between cells, and equation (1) implies that auxin production and degradation are proportional to cell volume. This will play a role in our later discussion of sink-driven systems and meristem systems.

We now discuss the two generic systems, source- and sink- driven, that comprise the SAM and FM, and their observation (or not) of the requirements for canalization.

### Source-driven Systems

These are systems with (a) localised source(s) significantly stronger than production throughout the rest of the tissue. This is a crude approximation of the inner meristem, for which the primordium acts as a realistic source. Polar-in behaviour follows intuitively from the localized source combined with the PIN initiation being generated by diffusive flux (except perhaps for contrived situations that do not interest us here). Furthermore, our analysis of the polar-out requirement found that polar-out behaviour, and therefore canalization, is naturally and robustly canalized. Our simulations found this also. (The default parameter values used in our simulations are given in table 2 while non-standard values are given in table 3.).

**Table 2:** Default simulation parameters.

Parameter	Variable	Equations	Default	Units
square/cubic cell volume	*V*	1	1	*μm* ^3^
hexagonal cell volume	*V*	1	2.60	
wall area	*a*	1	1	*μm* ^2^
hexagonal wall area (3D only)	*A*	1	2.60	*μm* ^2^
auxin production (sink-driven)	*α*	1	0.2	*μmol μm^−^* ^3^ *hr^−^* ^1^
auxin production (other)	*α*	1	0	*μmol μm^−^* ^3^ *hr^−^* ^1^
auxin degradation	*β*	1	1.0	*hr* ^−1^
PIN production	*α_P_*	5	0	*μmol hr^−^* ^1^
PIN degradation	*β_P_*	5	1.00	*hr^−^* ^1^
diffusive transport coefficient	*γ*	2	1.00	*μm hr^−^* ^1^
PIN transport coefficient	*γ_P_*	2	40.0	*μm hr^−^* ^1^
flux-induced PIN coefficient	*κ*	5,6	1	*μmol μm^−^* ^2^ *hr^−^* ^1^
reference flux (density)	*J_ref_*	6	1	*μmol hr^−^* ^1^
maximum PIN available per cell	*P_T_*	6	1	*μmol*
reference PIN available per cell	*ρ_ref_*	5,6	1	*μmol*
feedback exponent	*σ*	5,6	2	1
PIN cutoff (non-competitive only)	NA	NA	10	*mol μm^−^* ^2^

**Default model parameters.** These are the values used throughout the paper except where stated otherwise. Cell volumes and areas vary around their default values by a small margin on randomized tissue grids. “NA” means “not applicaple. The PIN cutoff does not appear explicitly in any equations. See also table 3.

**Table 3:** Exceptional simulation parameters.

Figures	Variable	Value	Units
15	*V*/*a*	25–45/2–5	*μm* ^3^/*μm* ^2^
18	*V*/*a*	95–300/6–60	*μm* ^3^/*μm* ^2^
3,4*	*α/β*	0.4/0.0	*μmol μm^−^* ^3^ *hr^−^* ^1^/*hr^−^* ^1^
11C	*α/β*	0.08/0.4	*μmol μm^−^* ^3^ *hr^−^* ^1^/*hr^−^* ^1^
14*	*α/β*	0.5–0.9/0.0	*μmol μm^−^* ^3^ *hr^−^* ^1^/*hr^−^* ^1^
18	*γ/β_P_*	0.05/5.00	*μm hr^−^* ^1^/*hr^−^* ^1^
3,4	*γ_P_*	1.0	*μm hr^−^* ^1^
15,18	*κ*	0.2	*μmol μm^−^* ^2^ *hr^−^* ^1^
7–13,16,17, 18	*σ*	1.5!,(1.0,1.1),1.1,(1.5,2.0),1.5	1
8C,16,17	PIN cutoff (non-competitive only)	5.0,20,20	*mol μm^−^* ^2^

**Exceptional model parameters.** Table of parameters and figures in which the parameter values differ from those given in table 2. An asterisk (*) indicates variation in the relevant variable. An exclamation mark (!) indicates that the qualitative result is relevant over a range of values. In both cases the reader is referred to the figure’s caption.

**Figure 5:**
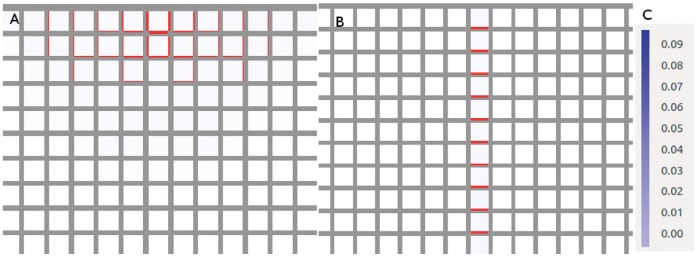
Source-driven systems. These simulations are run on a square grid much larger than shown here (see other figures with square grids) but we have zoomed in on the PIN distribution around the source for clarity. The top-centre cell of the grid is a source. (A) Linear feedback (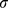 *= *1.0). (B) 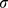 *= *1.5. This result is representative of similar simulations for any 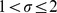. The length of the vascular strand is sensitive to changes in the background auxin degradation and the strength of the source. (C) The colour bar indicates the auxin concentration in *µmol* *µm^−^* ^3^ in this and all remaining simulations.

For non-competitive models the steady state changed from diffuse PIN to canalized PIN when 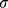 changes from 1.0 to 1.1. All simulations had a lone, canalized strand as their final state with super-linear feedback. For very low degrees of super-linearity (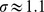 with our parameters) there was a tendency to pass through a transient diffuse stage, but otherwise a lone strand grew directly (See figure 5).

**Figure 6:**
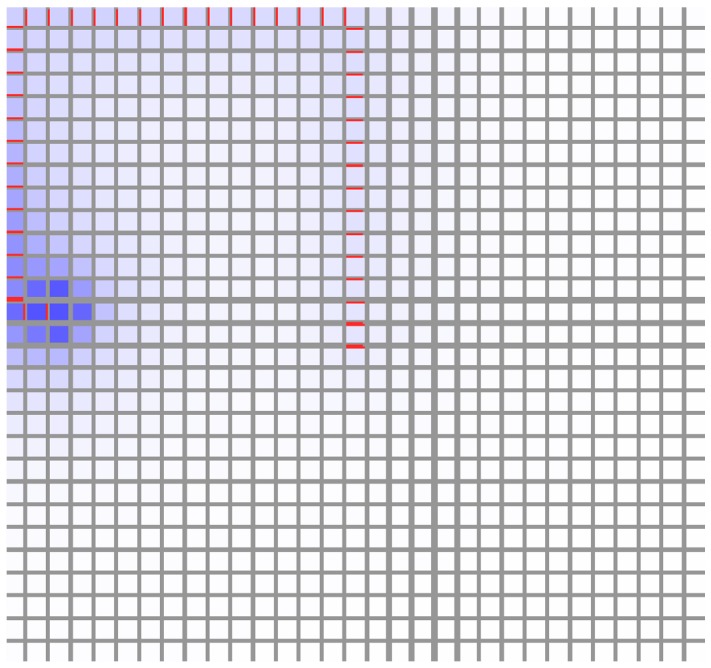
Source-driven system with the source at the centre of the tissue. Despite perfect square symmetry, a vein forms in one direction only. This is an example of spontaneous symmetry breaking.

A notable observation also made in [12] is that, unlike the sink-driven systems, we could not find steady states of the source-driven system with vascular strands in multiple directions, in the absence of saturation. Indeed, when the source was in the centre of the tissue with no features to induce a direction then, in the absence of saturation, there was either no significant strand formation at all, or a strand grew in one direction only. In a realistic tissue this direction would be given by the irregularities in the tissue, but on a regular grid it is an example of spontaneous symmetry breaking (SSB), *i.e.* choosing one direction in preference to others despite complete symmetry between them, as shown in figure 6. This is common in physical systems, *e.g.* a ferromagnetic material will spontaneously magnetize in a certain direction when it cools below its Curie temperature, with nothing other than thermal fluctuations to push it in that direction. The *dynamics* of the material are perfectly isotropic but its *lowest energy state* is not. Although counter-intuitive, this is to be expected from our earlier discussion on polar-out behaviour. This result also held on grids of hexagonal cells and on three-dimensional grids of cubic cells, although we sometimes observed long-lived transient multi-wall distributions. (Transient states and metastability are beyond the scope of this paper.).

#### Irregular tissues

**Figure 7:**
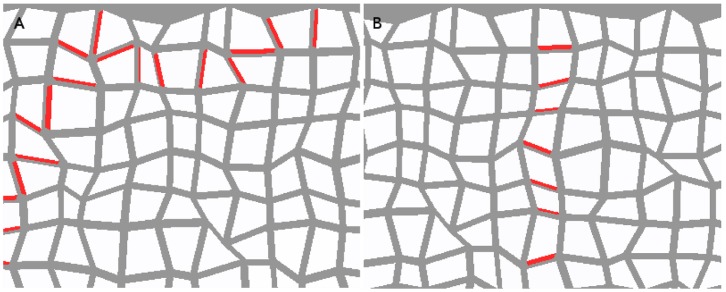
Non-competitive, source-driven model on a randomized tissue grid. These simulations were run on a tissue generated by randomizing the vertex positions of a square grid. As in figure 5, we have zoomed in on the region surrounding the source, for clarity. Both distributions are canalized. They both have 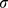 *= *1.5 but the same qualitative result was found for all 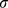 *>*1, and also for competitive models. (A) In the flux-based simulation the emerging vascular strand twists and turns in response to cell irregularity, while in (B) the flux density-based simulation grows in a straight line, demonstrating that use of the flux-density buffers the PIN dynamics against irregular cell geometries.

Simulations of source-driven models on irregular tissue grids grew vascular strands from the source which twisted and turned in random directions, but otherwise resembled the corresponding simulations on a regular tissue grid (see figure 7). This is reminiscent of simulations in Bayer *et. al.* [13] where the strand in a realistic SAM twisted and turned in response to cell geometry, requiring an additional mechanism to bias strand growth in the desired direction. While there is experimental evidence [13] for a mechanism guiding vein growth towards existing vasculature, we were able to grow straight vascular strands with flux density-driven models. The corresponding results also emerged on three-dimensional tissues (not shown).

The behaviour of the source-driven system was therefore seen to be robustly canalized against parameter changes and cell geometry. The effect of cell irregularity was to make the strands twist and turn randomly, which could be largely negated by replacing the role of flux in the PIN dynamics with flux density 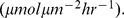.

### Sink-driven Systems

These are systems in which auxin is slowly produced and degraded throughout the tissue except for a particular cell (or group of cells), which degrades auxin at a relatively rapid rate. As mentioned earlier, sinks are typically modelled as either perfect or realistic. It is known experimentally that primordia, which form effective sinks in the L1 of the SAM and FM, have a higher level of auxin than the surrounding tissue, so they cannot be realistically modelled as perfect sinks. Our simulations included both perfect and realistic sink models, the latter of which readily replicated the auxin peak found at primordia.

As shown by Stoma *et al.* [5], a sink-driven system whose PIN allocation is flux-mediated will show qualitatively different final distributions of PIN according to whether PIN production varies linearly or quadratically with the flux. In the former case a diffuse, homogenous distribution of PIN pointing towards the sink is always produced, while the latter may produce well-defined paths of polarised PIN believed to form the foundation for vascular strand formation [17].

**Figure 8:**
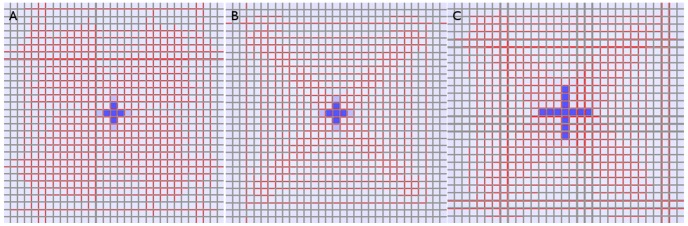
Sink-driven simulations. In all three cases the sink is an auxin maximum. PIN is ubiquitous and oriented towards the sink (central cell). (A) This simulation has a linear feedback function (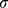 *= *1.0) and the polar-out requirement is not obeyed. (B) This simulation has feedback exponent 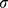 *= *1.1. Here the polar-out requirement is obeyed, except where the PIN cut-off is met. (C) This is the same as (B) except that the PIN concentration cap has been halved to 5 *µmol* *µm^−^* ^2^. The build-up of auxin around the sink (centre) is larger in extent and the polar-out requirement is violated more often due to PIN saturation occuring at lower PIN concentrations.

**Figure 9:**
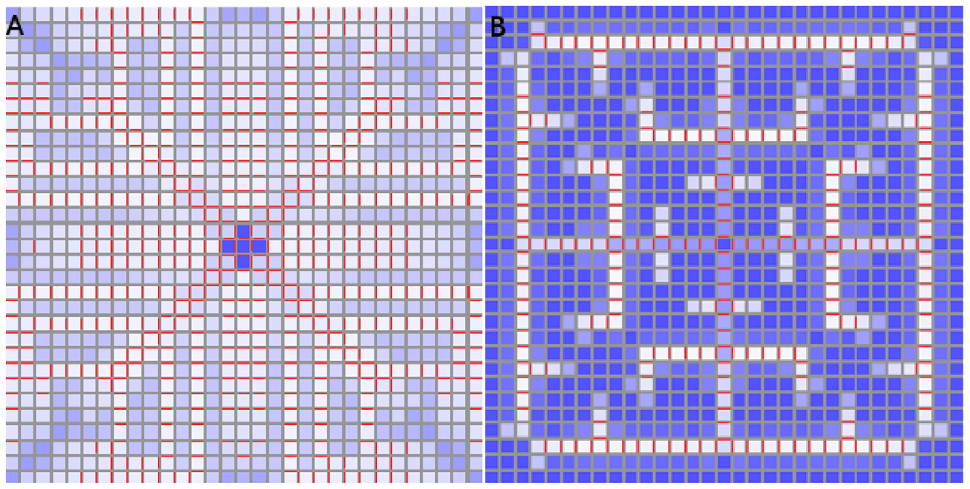
Sink-driven system with different feedback exponents. A higher 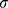 value generates a less branched pattern whose vascular strands are much better defined. (A) 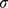 *= *1.5, (B) 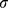 *= *2.0.

We now present a study of 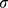 values intermediate between one and two (see figures 8,9), which found that the transition in these systems occurs gradually over a range of values. Specifically, although the polar-out condition is obeyed whenever the feedback is super-linear, multi-neighbour behaviour fades out gradually with increasing super-linearity.

#### Two-dimensional simulations

We ran several simulations on a square lattice of 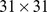 cells where the central cell functioned as either a perfect or a realistic sink. The differences between the perfect and realistic sink results were negligible except for the auxin concentration in the sink which is zero in the former case and elevated in the latter. (The reader is reminded that the parameter values are given in tables 2 and 3.).

As expected from previously published results [5], the PIN distribution for a linear feedback function 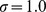 gave a diffuse PIN distribution (figures 8A, B), with an accumulation around the primordium for both realistic and perfect sinks. The rise in auxin was significant.

There was an important qualitative difference at 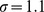. Here the PIN was still ubiquitous but there is only one face in each cell expressing PIN, except along the diagonal. These exceptions were due to the upper bound placed on PIN concentrations, which we verified by checking that the PIN concentrations in these cells equal the upper bound in at least one and usually both walls that show PIN. The incidence of multiple walls expressing PIN could be reduced by increasing the upper bound on PIN concentration (not shown). This is to be expected from our discussion of the polar-out requirement, which demonstrated that even a minute amount of nonlinearity is sufficient to ensure that cells express PIN in only one wall.

The very sharp auxin peak at the sink could be spread by imposing a lower cap on the PIN concentration. When the cap was lowered from ten to five 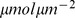 then the peak was spread (see figure 8C).

For 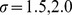 the patterns are best described as highly branch venation patterns, where the quadratic case is less branched and its vascular strands are better defined (see figure 9).

We ran corresponding simulations for the competitive PIN model. We first confirmed that the auxin levels are higher in the vascular strand than in the surrounding tissue [4]. We also found a very similar transition from diffuse to canalized PIN, but with a reduced degree of branching due to the higher concentration of auxin in the vascular strands. A similar transition was also observed (not shown) with changes in the auxin production rate 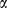, where higher 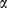 corresponded to heavier branching. This is consistent with our discussion of the polar-in condition.

#### Three-dimensional simulations

**Figure 10:**
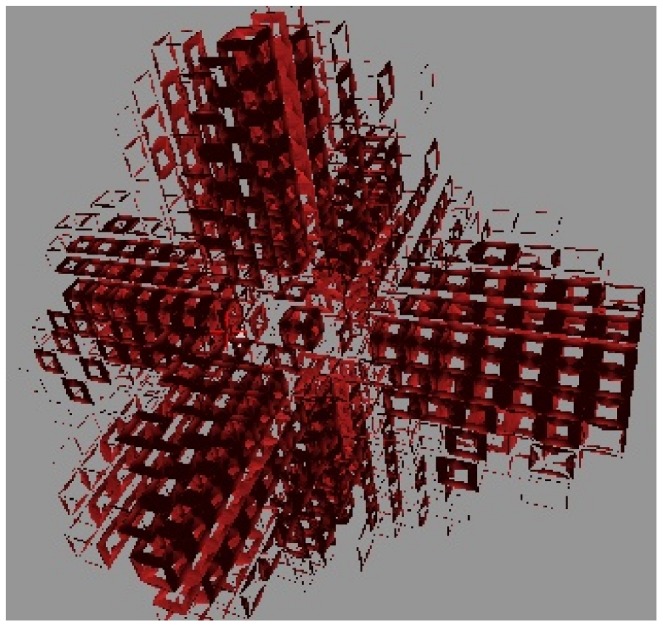
PIN distribution for a three dimensional sink-driven system. The display of cells has been repressed for the sake of clarity. This snapshot was taken while the simulation was still incomplete but one can already see the excessive branching from the initial veins. This simulation went on to almost fill the entire tissue with sub-lateral veins (not shown). the sink is in the exact centre of the tissue. Similar results were found for the competitive model.

We have also simulated three-dimensional sink-driven systems, in which the sink lies at the centre of a 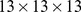 grid. We found that the transition from diffuse to canalized does not run the same way (see figure 10). It appeared to do so in the early stages of the simulation, but eventually the PIN came to be expressed throughout the tissue. The observed pattern in both competitive and non-competitive models was that many of the cells in a vascular strand acted as a sink in the orthogonal plane intersecting the strand at that point. Although there was some interference between adjacent planes, it was not enough for the final state of the tissue to make a meaningful analogy with its two-dimensional counterpart. We ran simulations with lower auxin production and PIN-related parameters and even tried increasing 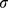 to values up to and including three, but the simulation would either have no PIN production or eventually produce it over most of the tissue grid.

We concluded from this that a three-dimensional tissue is unlikely to use a sink-driven system to form vascular strands.

#### Analysis of branching suppression

That any amount of super-linearity is sufficient to enforce the polar-out condition was also observed in the sink-driven simulations, where exceptions were due to the cut-off value imposed on the PIN concentrations.

We have also seen that enforcement of the polar-in condition arises from competition between aspiring PIN-expressing walls, and that this is strongly dependent on tissue geometry and also auxin- and PIN- related parameters. The above section on two-dimensional simulations demonstrated the variation of canalization in response to changes in the flux feedback exponent 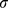. We also found similar variations in response to changes in auxin and PIN production (not shown). For example, changing auxin production to 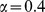 in the simulations of figure 9 destroyed their neatly branch canalization. Alternately, lowering 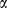 in a sink-driven system was found to have a similar effect to raising 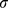 when the degree of super-linearity was relatively low (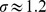). The effect of cell geometry is primarily due to a cell’s auxin production being proportional to the cell’s volume, which follows from equation (1) describing the time evolution of auxin *concentration*. If all walls have unit area, as they do in all our sink-driven simulations, then the square and cubic cells have unit volume while hexagonal cells have a volume of 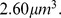.

**Figure 11:**
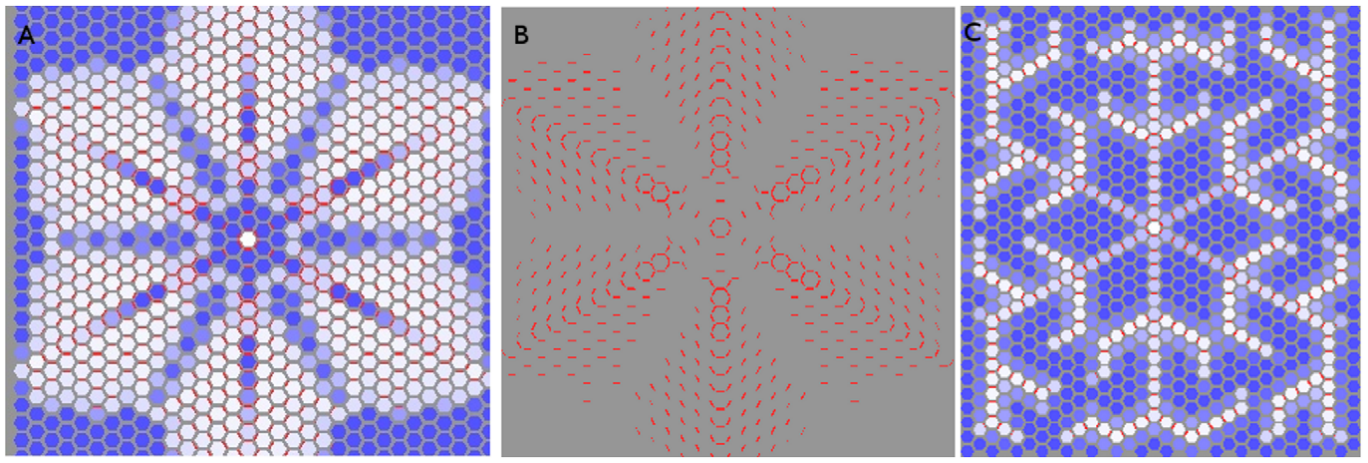
Non-competitive sink-driven model on tissue grid with hexagonal cells. In all three cases the sink is perfect and in the centre of the tissue. The walls have an area of one *µm* ^2^ and the cells a volume of 2.60 *µm* ^3^ *:* (A,B) The simulation is run with the same auxin-related parameters as on the square grid (figures 8,9). The PIN distribution is initially canalized, but with considerable multi-neighbour behaviour away from the centre. The cells in (B) are suppressed to show the PIN distribution more clearly. These pictures do not represent the final state of the simulation. We stopped it here because edge effects disturb the distribution in a way not relevant to the present discussion. (C) The auxin-related parameters *α, β* have been altered to compensate for the larger cell volumes. The resulting PIN distribution is highly analogous to 9B.

To show this we ran sink-driven simulations on a 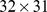 grid of hexagonal cells. The first used the exact same parameters as in figure 9B, while the second reduced 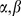 by a factor of 2.6. The result is clear to see in figure 11. Keeping the same auxin-related parameters resulted in frequent violation of the polar-in requirement while adjusting these parameters for cell size produced a result highly analogous to that of 9B.

**Figure 12:**
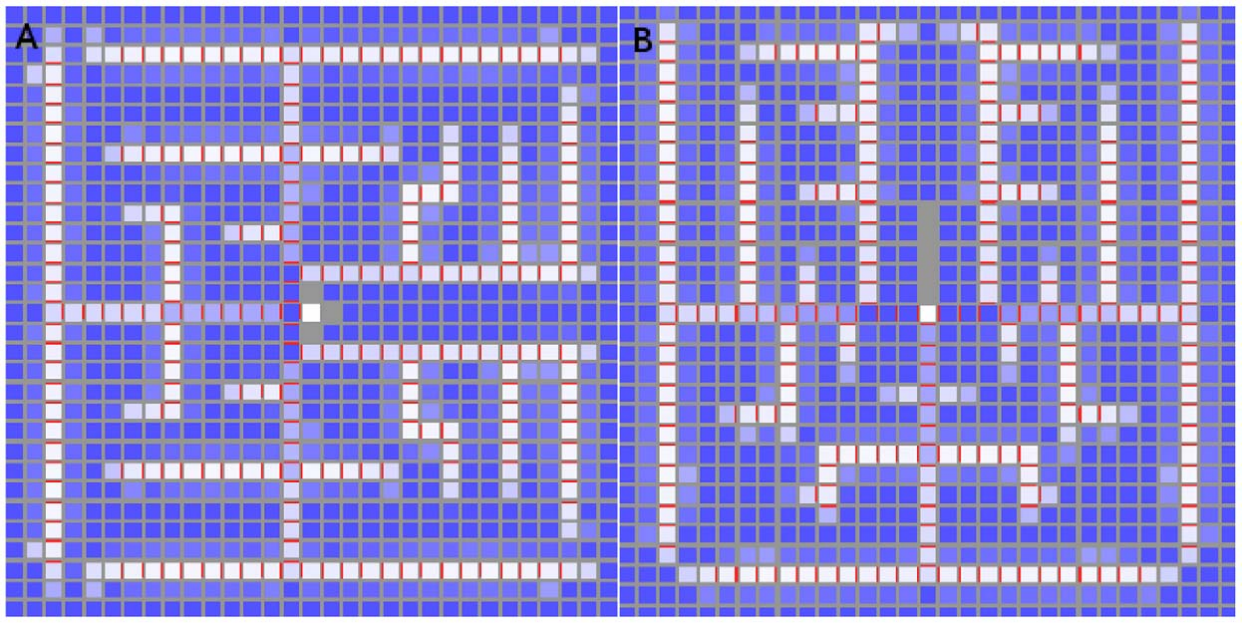
Sink-driven simulation with neighbours removed from the central sink. While allowing for the sink in these simulations being perfect, they should be compared to figure 9 B. (A) Neighbours were removed from the central sink. Additional laterals branched from the vein in order to get around the gaps. (B) A vein was cut out when it was five cells long. The two adjacent veins branched in response.

Inspection of figure 11A,B indicates that with hexagonal cells the secondary strands grew until they interfere with each other, suggesting that branching is blocked by the presence of other strands. We tested this with a series of simulations in which a vascular strand was either prevented or removed. (See figure 12). In each case the closest remaining strands showed altered branching patterns while those further away were typically unaffected.

**Figure 13:**
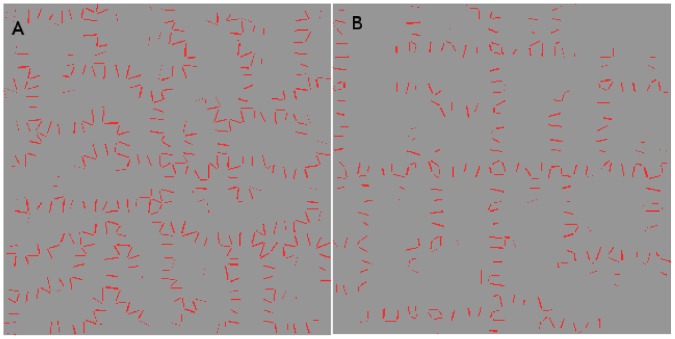
Non-competitive model with quadratic feedback on a randomized tissue grid. The simulation on the left is flux-driven while that on the right is flux density-driven. These PIN allocation are canalized but the left-hand one is very messy. The same qualitative result was found in the competitive model (not shown). The tissue is the same one used in figure 7 but the cells have been repressed for clarity.

We ran additional simulations on a randomized tissue, where the corners of the cells were randomly displaced by small amounts. The strand formation was unmistakable (see figure 13). It showed considerable twisting and additional branching in the flux-based model, but a flux density-based model is more reminiscent of its counterpart on the regular tissue grid. This indicates that the loss of polar-in behaviour is sensitive to cell geometry rather than irregularity.

We now discuss another appealing hypothetical mechanism for the emergence of polar-in behaviour and the limitation of vein branching. Perhaps neighbouring cells which start pointing PIN toward a cell inhibit other cells from doing so, by raising the auxin level in the receiving cell and weakening the diffusion driven flux needed to stimulate initial PIN expression in other neighbours. Thus any neighbour with weaker PIN expression is further disadvantaged while those with strong PIN expression are only weakly affected. If the diffusion is sufficiently weakened early enough the neighbours with lagging PIN may never have sufficient flux to generate significant amounts. This is very similar to the competitive canalization mechanism proposed by Prusinkiewicz *et al.* [18] for the regulation of bud activation in plants. This mechanism was observed experimentally by Balla *et al.* [19] and found to emerge in the simulations of both [18] and later, Wabnik *et al.* [20]. This last example is a molecular model based on ABP1 and operates at a more mechanistic level than either the flux- or concentration- based models.

**Figure 14:**
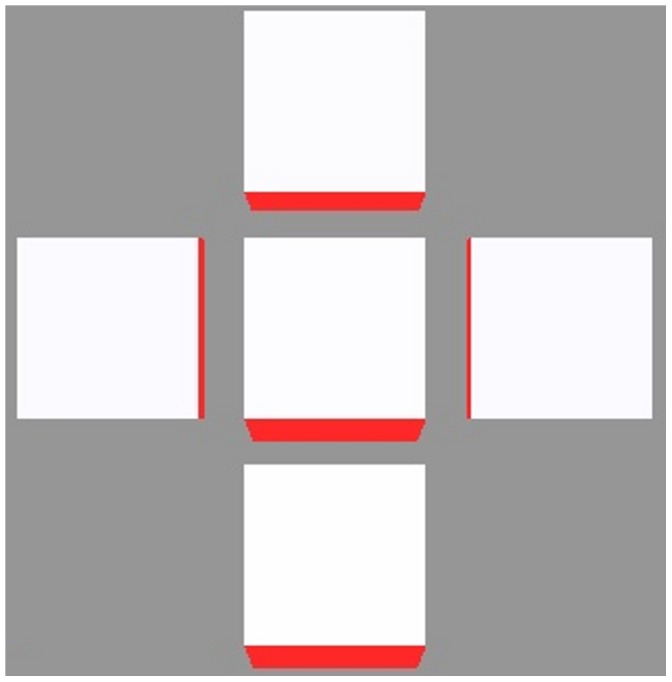
Demonstration that available flux alone accounts for PIN generation. The centre cell is due to be the next cell in a growing vein, represented by the perfect sink at the bottom with the strong PIN expression. The top cell has higher auxin generation than the other two, and the final PIN concentrations are proportional to the square of the auxin generation when 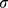 *= *2. Thus conformity to the polar-in requirement is largely unaffected by dynamics within the receiving cell.

To test this we ran simulations, illustrated by figure 14, designed to replicate the region at the tip of a growing vascular strand in a sink-driven system. The bottom-most cell is a perfect sink and represents the established vascular strand removing the auxin from the site. (The PIN visible at its lower face is intended to convey this, although it had no practical effect in this simulation.) The central cell is due to be the next cell in the growing strand, and the point of interest was the expression of PIN by its neighbours. In order to accurately reflect the influence of flux, all auxin degradation was turned off except for the perfect sink at the bottom, and auxin generation is limited to the other extreme cells. The above hypothesis predicted that if the top cell had a sufficiently higher auxin production rate, then it would prevent the other neighbours from expressing PIN toward the central cell.

We ran this simulation always with quadratic feedback, where the effect would be strongest, over a wide range of different auxin generation rates. Without fail, the final state had all three neighbours expressing PIN in proportion to the square of the auxin production regardless of the differences in production rates. This is predicted by a straightforward inspection of the PIN generation rate, and we concluded that the dynamics of vascular strand branching are governed by the flux available to each neighbouring cell. The absence of anything resembling competitive canalization is not mysterious. One factor is that the sources are all turned on simultaneously. Another is that the vein tip had a large PIN concentration at the beginning of the simulation, which maintained a low auxin concentration there by removing the auxin more quickly than the growing PIN concentrations in neighbouring cells could flux it in. This is in contrast to [18] and [20] whose canalized PIN distributions emerged from sources and had elevated auxin concentrations.

A vascular strand will branch if more than one of its neighbouring cells have a sufficient auxin supply to stimulate PIN in its direction. This depends on the geometry of the cells, the topology of the tissue, the positioning of sources and sinks, auxin and PIN production throughout the tissue, and the presence of other vascular strands. It also depends on the degree of nonlinearity in the flux feedback function, with greater super-linearity requiring a larger auxin flux to generate PIN, but also yielding a higher PIN concentration when PIN does establish, which transports auxin away more efficiently.

The most important message of this section is that canalization in the sink-driven system is far from robust, conformity to the polar-in requirement being compromised by low super-linearity and cell geometry.

### Digitized L1

We recreated the sink- and source- driven systems on a digitized L1. This was biologically relevant only for the sink-driven system, since the L1 is sink-driven and not source-driven, but our purpose was to study the effect of realistic cell geometry on the above results.

#### Sink-driven systems

**Figure 15:**
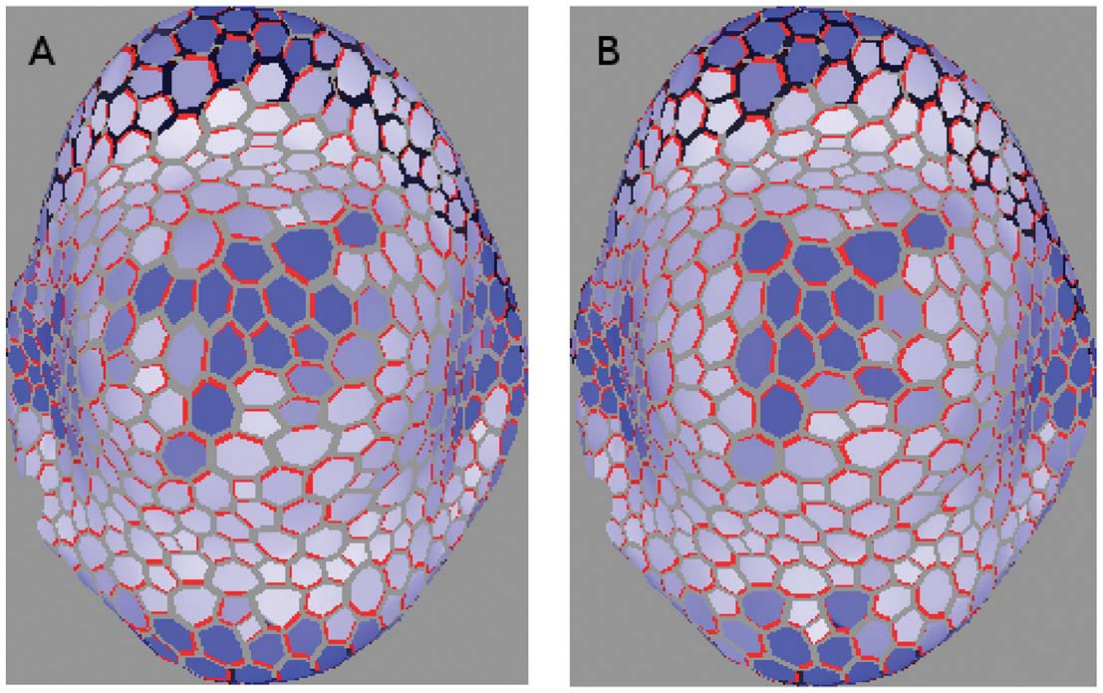
Competitive flux- (left) and flux density- (right) driven model with quadratic feedback on a digitized L1. The centre of the tissue and of each of the lobes is centred on a realistic sink, each of which has an auxin build-up around it. The PIN has a diffuse distribution with no regard for the polar-in requirement and occassional violation of polar-out due to saturation effects.Qualitatively similar results were found for the non-competitive model (not shown). Cell volumes ranged from 25 to 45 *µm* ^3^ while wall areas ranged between two and five *µm* ^2^.

Canalization was effectively eliminated for a range of parameter values (see figure 15). Specifically, there is little or no respect of the polar-in requirement. This was an important difference between the square and hexagonal tissue grids and should not be surprising since the digitized cells are closer to hexagonal than square.

#### Source-driven systems

As with the irregular tissue, flux-based simulations generated veins that twisted and turned in sympathy to local tissue conditions, while the veins of flux density-based simulations were straighter (not shown) with some bending due to surface curvature. Hence the qualitative results of previous sections were preserved on the digitized L1.

### Meristem Systems

These are systems whose top layer is a sink-driven system while the rest of the tissue forms a source-driven system, by analogy with real SAMs and FMs (see figure 1). The sink of the L1 layer transports auxin to the interior like the primordium in a real SAM, hence it is a realistic sink in the L1 and a realistic source for the interior. PIN-mediated flux between the L1 and the rest of the tissue is forbidden, except for the primordium. We were unable to generate the desired distribution of L1 PIN pointing towards the primordium and then growing a vascular strand into the interior without this condition, for which there is experimental evidence [21]. To replicate the vascular strand connecting to existing vasculature, and to avoid auxin building up in the interior, we also placed a perfect sink in the interior where a growing vascular strand might intercept the existing vascular bundle. However, our simulations did not include a mechanism for finding existing vasculature.

A unified model requires parameter values, especially for 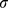, that yield a diffuse PIN pattern in the L1 and a canalized one in the interior. We have seen that super-linearity is both necessary and sufficient for canalization in the interior, whereas diffuseness can be achieved in the L1 provided that either the degree of super-linearity is sufficiently low or the flux (density) is sufficiently high for a given tissue geometry.

In two-dimensions the L1 is one-dimensional, so it is not possible to differentiate between diffuse and canalized PIN distributions. A unified model therefore can only be demonstrated in three dimensions.

**Figure 16:**
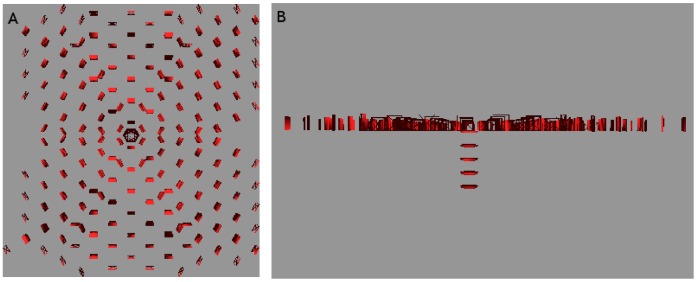
Meristem system on a three dimensional tissue consisting of layers of hexagonal prisms. As required the top layer, representing the L1, has a diffuse PIN allocation pointing towards the primordium (left), from which a straight line of PIN leads down to the sink at the bottom (right). The cells have been repressed for clarity. Auxin production is limited to the L1 with the value *α = *0.2 *mol* *µm^−^* ^3^ *hr^−^* ^1^. Auxin degradation in the L1 is *β = *1.0 *hr^−^* ^1^ *;* while that in the interior is *β = *0.4 *hr^−^* ^1^ *:* Hexagonal faces have an area of 2.60 *µm* ^2^ and the cells’ volume is 2.60 *µm* ^3^ *:*

**Figure 17:**
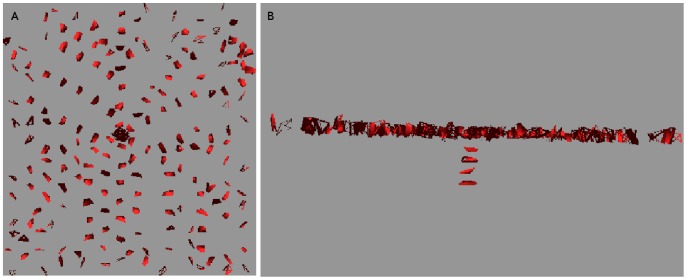
Same as figure 16 except that the tissue grid has been made irregular by small random displacements of the cells’ vertices. A dual PIN allocation is again observed.

In order to take advantage of the effects of higher numbers of cell walls we performed three-dimensional simulations on tissues comprised of layers of hexagonal cells. As can be seen in figures 16, 17 the L1 contains a diffuse spread with little compliance to the polar-in requirement, on both regular and irregular tissues. This result held for both competitive and non-competitive models. It can be replicated on cubic tissue grids (not shown) but is less robust since 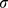 must be kept lower in order to obtain the diffusive L1 distribution while higher 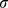 makes it easier to make the vascular strand grow straight.

**Figure 18:**
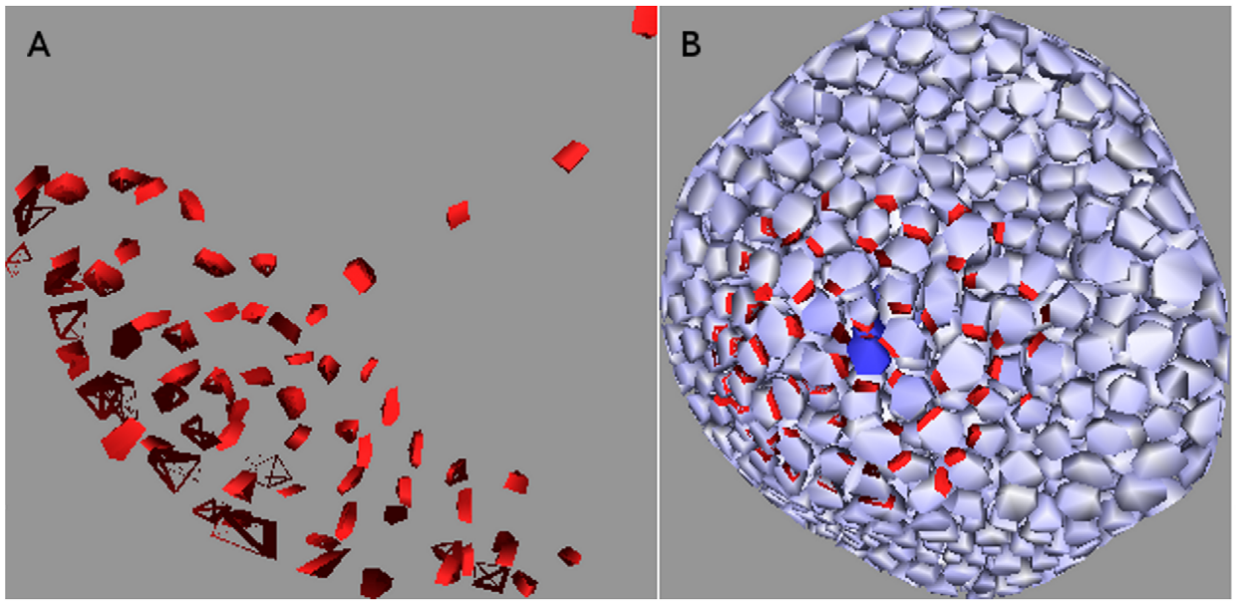
Competitive model with 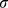 *= *1*∶*5 on digitized oral meristem. The cells are repressed for clarity in the left-hand picture. A small region on the surface produces auxin like an L1, with its cells pointing PIN toward the primordium at the centre, while a canalized mid-vein grows into the interior. The right-hand side indicates a rise in auxin is visible at the primordium. Auxin production is limited to the L1 with the value *α = *0.1 *mol* *µm^−^* ^3^ *hr^−^* ^1^. Auxin degradation in the L1 is *β = *1.0 *hr^−^* ^1^, while that in the interior is *β = *0.4 *hr^−^* ^1^. Cell volumes range from about 95 *to* 300 *µm* ^3^ with wall areas ranging from six to about sixty *µm* ^2^.

We generated the dual PIN distribution on a digitized floral meristem. As with the digitized L1 used earlier, the point was to be working with realistic cell geometries rather than to completely replicate biological reality. In figure 18 we show the results of a competitive simulation with feedback exponent 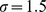. The right-hand side of 18 clearly shows the L1, to which all auxin generation is restricted, with the required diffuse PIN distribution, while the cells are suppressed on the left-hand side so that PIN allocations inside the tissue may be seen. The line of strong PIN allocation growing out from the centre of the diffuse distribution is clear.

While the reproduction of a PIN distribution that is canalized in the interior of the meristem but diffuse on its surface was straightforward, it was difficult to avoid transient states that render the simulation biologically irrelevant. Specifically, although the final state cannot help be canalized in the interior of the meristem in the absence of saturation effects, temporary branching can and sometimes does occur in source-driven systems. Since biologically the vascular strand grows neatly into the interior, simulations in which the interior transitively fills with PIN are not biologically relevant regardless of their final state.

The mechanism behind this is easy to understand. Source-driven branching in the absence of saturation occured in the early stages of PIN allocation in a cell, when the diffusive component of the auxin flux was not yet dominated by PIN-enhanced flux. The obvious remedy was to reduce the diffusive contribution to the flux without directly affecting the PIN component, and the best way to do this was to reduce the permeability of the cell walls. This worked well and allowed the biologically relevant generation of figure 18 from both competitive and non-competitive models with a variety of values for 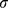.

We were able to generate, on systems that reflect the basic geometry of a SAM or FM, steady-state PIN distributions that imitate the biological allocation of PIN in the region of a primordium [1], [2]. Specifically, the two-dimensional top layer had one cell playing the role of primordium by being the only L1 cell capable of active transport to the interior, although we did not block diffusion between sections. These distributions had diffusive PIN distributions pointing towards the primordium in the L1, which seeded a strictly canalized strand into the interior of the meristem. The three-dimensional tissues were a regular tissue of layers of hexagonal cells, a randomized form of this, and a digitized floral meristem.

## Discussion

We have studied the occurence in both flux- and flux density- driven PIN models of both diffuse PIN distributions and vascular strand-forming ones. Sensitivity to parameters and cell geometry strongly depends on the distribution of sources and sinks. These different source/sink distributions were generically characterized as sink-driven and source-driven. The sink-driven system has uniform auxin dynamics everywhere except in a localized cell, or localized region of cells, which act as a sink. Conversely, the source-driven system has uniform auxin dynamics everywhere except in a specific cell, or region of cells, which act as an auxin source. We considered both a competitive model, where the walls must compete for a limited supply of PIN, and a non-competitive model. Our qualitative results are summarised in table 1.

Source-driven systems make the transition from diffuse to canalized with only the smallest amount of super-linearity and regardless of cell geometry, but the sink-driven systems make this transition slowly with changes in the exponent of the feedback function. They are also sensitive to changes in cell geometry, to the point that canalization vanished on some digitized tissues even with quadratic feedback.

On a distorted grid the degree of canalization is similar to a regular grid. The chief differences are that the strands are not straight, and in sink-driven systems there is an increase in the amount of branching. These effects are largely negated when flux-density is used instead of flux, which is readily understood as flux-density canceling the effects of differing face areas.

This permitted our final simulations of the SAM as a juxtaposition of a sink-driven and a source-driven system. Its top layer was considered the L1, and produced and degraded auxin everywhere except in the centre cell, which was designated the primordium. The rest of the tissue simply degraded auxin at a mild rate except for a perfect sink which was the destination of the vascular strand. PIN-mediated auxin transport from the L1 to the interior was strictly restricted to the primordium. This is consistent with experiments finding [21] that auxin transport from the L1 to the interior is greatly inhibited.

Both our own and previous [16] (also see Text S1) analyses of source-driven systems found a distinct inclination toward canalization, in that the stable PIN configurations consist of significant PIN expression being limited to one wall within a cell for any non-linear feedback function. Indeed, so strong is this inclination that a source in the centre of a symmetric tissue could spontaneously break the directional symmetry by choosing a random direction to grow the vascular strand. This phenomenon, well-known in physics as spontaneous symmetry breaking (SSB), is when a system “chooses” a configuration, or “ground state” in physics terminology, which violates a symmetry of the dynamics because no symmetric configuration is stable. (Physicists say that the system breaks the symmetry in order to lower its energy.) Despite considerable variation in parameters, we never observed the source-driven system to grow strands in more than one direction from the centre in a two-dimensional system. We did observe it in three dimensions, but we also observed SSB and believe that the absence of it in some simulations is a numerical artifact.

We also performed three-dimensional simulations of source- and sink- driven systems. It is a common assumption in the literature (eg. [13]) that three-dimensional tissues can be simulated with two-dimensional cross-sections. This approximation works well enough for the source-driven system but is problematic for sink-driven systems.

While source-driven systems seem to be insensitive to dimensionality, which follows from our discussion of Feugier’s linear stability analysis (see Text S1), the sink-driven case is more interesting. The linear feedback function yields the corresponding result, while the quadratic case initially grows vascular strands but branches them so often that the final configuration is full of PIN. Veins would initially form but have so many laterals that the tissue would eventually fill with them. Varying parameters either stopped PIN production altogether or made little impact on the final result. So, for sink-driven systems, the approximation can serve as a guide, especially for short-term dynamics, but should be treated warily when considering the final state of the tissue.

One criticism of flux-based models is the apparent lack of any means for the cell to sense the flux through its walls [3], [22], leading to the postulation of gradient models [23], where cells measure the auxin gradient across themselves, as a realistic approximation. In our view there is nothing mysterious about cells measuring the efflux of auxin through each wall. Auxin requires specialized effluxing proteins at the wall in order to efflux effectively. Furthermore, whenever an auxin molecule is transported by such a protein that protein must be recycled, since it is capable of transporting against a gradient without consuming ATP. This could potentially give accurate information to the cell about the rate of auxin efflux, measured as PIN endocytosis, additional to the background rate, due to gene networks such as those studied in [24], [25], [26], [27]. While the actual gene/molecular network calculating the desired PIN generation and exocytosis rates remains unknown, it is easily conceivable that one should exist. Indeed, a feedback mechanism for diffusive auxin flux through walls based on proton flux has been proposed and modelled by Steinacher *et al.* [28]. That such a network would distinguish between walls is also feasible. The ROP network, for example, is known [29] to regulate PIN and microtubule polarization at the cell wall.

Furthermore, since the auxin flux’s contribution to PIN recycling is proportional to the flux, the dynamics of PIN *density* 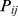 should depend on flux *density* 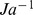, and we have seen that making this substitution greatly improved PIN distributions on both randomized and realistic tissues. This is especially true for vascular strand production in source-driven systems.

Another criticism of flux-based models [13] is that even though they can maintain flux against a gradient [5], they still require diffusive flux to stimulate PIN generation in the first place. This requires an initial drop in auxin concentration at the primordium, which is not experimentally observed. It is also difficult to replicate the observation [13] of PIN pointing towards the L1 and vascular strands with elevated auxin concentrations. Kramer [30] has shown that this problem can be addressed by including known influxers like AUX, which are neglected in many models. PIN generation can be stimulated by any form of flux in this model, and we suggest that the inclusion of influxers whose expression is stimulated by high auxin concentrations could naturally induce the initial L1 directed PIN orientations which Bayer *et al.* [13] interpret as concentration-based model effects. This line of enquiry will be explored in the near future.

We have shown that the contrasting modes of PIN expression in the different parts of the SAM and FM may be due to the contrasting sink versus source role played by the primordia. It follows that a unified model based on flux can explain auxin transport in both the L1 and the inner tissue. However, mixed models have also been shown to be plausible candidate mechanisms [5], [13] (in the sense that they also explain the emerging patterns). These theoretical insights have progressively bound the space of regulation mechanisms that can reproduce biologically plausible PIN patterns in tissues. More knowledge is now needed on the detailed mechanisms regulating PIN synthesis and membrane allocation at cell resolution in order to distinguish between the candidate models.

## Supporting Information

Text S1 **Summary and extension of Feugier’s analysis.**
(PDF)

Text S2 **Super-linear expansion.**
(PDF)
