# Supplementary material for: The Flux-Based PIN Allocation Mechanism Can Generate Either Canalyzed or Diffuse Distribution Patterns Depending on Geometry and Boundary Conditions
Source: PLoS One. 2013 Jan 28;8(1):e54802. doi: 10.1371/journal.pone.0054802 (PMC3557273; doi:10.1371/journal.pone.0054802)
Supplement: Text S1 — Summary and extension of Feugier’s analysis. (PDF) [file pone.0054802.s001.pdf]

Supporting Information Text S1

Summary and extension of Feugier's analysis

We begin with a brief overview of the linear stability analysis of steady states of auxin concentration and the PIN concentrations of two walls, performed by Feugier [1].

Consider a uniform cell with auxin concentration A, and PIN concentrations P_1, P_2 in two of its walls (walls are assumed throughout this analysis to be of unit area) and a fixed auxin influx/generation r, governed by the following dynamical equations:

$$\begin{aligned} \frac{dP_1}{dt} &= (AP_1)^2 + \alpha_P - \beta_P P_1, \\ \frac{dP_2}{dt} &= (AP_2)^2 + \alpha_P - \beta_P P_2, \\ \frac{dA}{dt} &= r - A(P_1 + P_2). \end{aligned} \tag{S1}$$

It is assumed in this analysis that the PIN-mediated flux dominates the diffusive flux, which can therefore be neglected. The reader should note that this choice of equations assumes that PIN-mediated flux dominates, which is, biologically, a very reasonable assumption although it may give misleading results at very low PIN concentrations. This system of equations has three solutions at steady state:

$$A = \frac{2r\beta_P}{4\alpha_P + r^2}, \quad P_1 = \frac{4\alpha_P + r^2}{4\beta_P}, \quad P_2 = \frac{4\alpha_P + r^2}{4\beta_P} \tag{S2}$$

$$\begin{aligned} A = \frac{\beta_P}{r}, \quad P_1 = \frac{r^2\beta_P - \sqrt{r^2\beta_P^2(r^2 - 4\alpha_P)}}{2\beta_P^2}, \quad P_2 = \frac{r^2\beta_P + \sqrt{r^2\beta_P^2(r^2 - 4\alpha_P)}}{2\beta_P^2} \\ A = \frac{\beta_P}{r}, \quad P_1 = \frac{r^2\beta_P + \sqrt{r^2\beta_P^2(r^2 - 4\alpha_P)}}{2\beta_P^2}, \quad P_2 = \frac{r^2\beta_P - \sqrt{r^2\beta_P^2(r^2 - 4\alpha_P)}}{2\beta_P^2} \end{aligned} \tag{S3}$$

His stability analysis based on the Routh-Hurwitz stability criterion found that for

$$r^2 < 4\alpha_P, \tag{S4}$$

equation (S2) is the only stable (and real) solution. In this scenario, $P_1 = P_2$ which are both very low in the regime where this solution is stable, implying minimal PIN and auxin efflux through those walls as expected at low r. P_1, P_2 do achieve increasingly higher values with increasing r under equation (S2), but such solutions are not stable.

When

$$r^2 > 4\alpha_P, \tag{S5}$$

it is equations (S3), obvious mirror images, that are stable, and only then when

$$\beta_P < r\sqrt{\frac{2\alpha_P - r^2}{4\alpha_P - r^2}}, \tag{S6}$$

which shall be true throughout our work. The stable solutions now describe a scenario where one PIN concentration is relatively high, increasing as a nonlinear function of r, while the other remains very low and decays exponentially with increasing r. Qualitatively, equations (S3) describe polar-out behaviour, with only one non-zero PIN concentration in the limit $r \to \infty$. Conversely, the difference between the two PIN concentrations approaches zero as equation (S5) approaches saturation.

The reader may object that the analysis only considers two walls when cells must possess at least three and usually posses several more. One may then argue that solutions exist where a subset of walls contains

elevated PIN, or even that the analysis breaks down altogether. The following argument extends these conclusions to cells with any number of walls by taking a cell in a stable steady state and considering its walls in pairs.

Lemma: If r is the net auxin production and influx into a cell in a steady state then the flux through a wall with PIN concentration P_i is

$$r\frac{P_i}{P_T}, \tag{S7}$$

where P_T is the total amount of PIN in the cell.

Proof: Follows from proportionality of flux and PIN concentration.

Lemma: PIN concentrations in walls of a cell at steady state have, at most, only two different values (assume walls have equal unit areas).

Proof: From the equation governing PIN dynamics (equation (3) in the main article) and the previous lemma,

$$P_i = \Big(\alpha_P + \Big(r\frac{P_i}{P_T}\Big)^2\Big)\beta_P^{-1}, \tag{S8}$$

whose solutions are

$$P_i = \frac{P_T^2}{2r^2}\Big(\beta_P \pm \sqrt{\beta_P^2 - 4\frac{r^2\alpha_P}{P_T^2}}\Big). \tag{S9}$$

These values depend on the unknown P_T, which itself depends on the proportion of walls with the higher and lower values. We can speak in this context of the cell's walls having either the high concentration or the low concentration. The polar-out condition corresponds to only one wall having the high concentration.

Theorem: In a stable steady state, there can be no more than one wall in a cell with the high concentration. In other words, either the PIN distribution is polar-out or all walls have the same concentration.

Proof: The combined flux through two walls of high PIN concentration is greater than that through two walls where one has the high concentration and one has the low concentration. We have from Feugier that two walls of different PIN concentration share a combined flux of more than $2\sqrt{\alpha_P}$. Therefore, the combined flux through two walls of high concentration is greater than $2\sqrt{\alpha_P}$. From Feugier, these two high PIN concentrations are therefore unstable and no more than two walls can have the high PIN concentration.

Corollary: In a stable steady state, a cell with n walls must be in the polar-out state if its auxin throughput is greater than $n\sqrt{\alpha_P}$, which is the critical flux marking the transition from equal PIN distribution to polar-out.

Proof: The first part is true because Feugier's stability analysis imposes an upper limit of of $2\sqrt{\alpha_P}$ on the flux through any two walls, or $\sqrt{\alpha_P}$ on the flux through any one wall. The second part is true because any pair of walls including the one with the elevated PIN concentration undergoes the transition when their combined flux is $2\sqrt{\alpha_P}$, corresponding to a total flux of $n\sqrt{\alpha_P}$

References

[1] Feugier FG (2006) Models of Vascular Pattern Formation in Leaves. Ph.D. thesis, Paris.
