# Supplementary material for: The Flux-Based PIN Allocation Mechanism Can Generate Either Canalyzed or Diffuse Distribution Patterns Depending on Geometry and Boundary Conditions
Source: PLoS One. 2013 Jan 28;8(1):e54802. doi: 10.1371/journal.pone.0054802 (PMC3557273; doi:10.1371/journal.pone.0054802)
Supplement: Text S2 — Super-linear expansion. (PDF) [file pone.0054802.s002.pdf]

Supporting Information Text S2

Super-linear expansion

Here we derive a result suggesting that a feedback exponent greater than one is sufficient to generate polar-out behaviour.

We take $\sigma = 1 + \epsilon$,

$$\frac{dP}{dt} = \kappa J^{1+\epsilon} + \alpha_P - \beta_P P, \tag{S1}$$

and the reader is reminded that we have set $J_{ref} = 1$. We expand $J^{1+\epsilon}$ in ϵ to get

$$J^{1+\epsilon} = J \exp \epsilon \ln J = J(1 + \epsilon \ln J + O((\epsilon \ln J)^2)). \tag{S2}$$

The relevant dynamics occur away from $J \approx 0$ so we can also expand the ln term around $\frac{J}{J_{ref}} = 1$,

$$\ln J \approx (J-1) - \frac{1}{2}(J-1)^2 + \cdots . \tag{S3}$$

J_{ref} clearly indicates the flux level above which taking a superlinear exponent of $\frac{J}{J_{ref}}$ will increase it, but decrease it otherwise. Substituting in these expansions, keeping only leading order terms, and taking PIN-mediated flux to be the dominant contribution, we get

$$\frac{dP}{dt} = \kappa(\epsilon(\gamma_P AP)^2 + A(1-\epsilon)\gamma_P P) + \alpha_P - \beta_P P, \tag{S4}$$

which is quadratic, *i.e.* of the same form as equations (S1) except for the $\kappa A(1-\epsilon)\gamma_P P$ term. Repeating Feugier's analysis with this equation would not be meaningful since the ln expansion is only valid around

$$\gamma_P AP \simeq 1. \tag{S5}$$

The additional term can be neglected when

$$\gamma_P A \ll \beta_P, \tag{S6}$$

in equation (S4). The condition (S5) then requires that P be very large, corresponding to the higher P value in the polar-out high r limit. Importantly, (S4) then reduces to equation (3) where $\kappa \to \kappa\epsilon$, indicating that if the conditions (S5,S6) hold then the PIN can be expected to evolve as in the quadratic case but with a weaker κ.
